# Supplementary figures and images for: Sensitizing drug-resistant cancer cells from blood using microfluidic electroporator
Source: PLoS One. 2022 Mar 8;17(3):e0264907. doi: 10.1371/journal.pone.0264907 (PMC8903260; doi:10.1371/journal.pone.0264907)

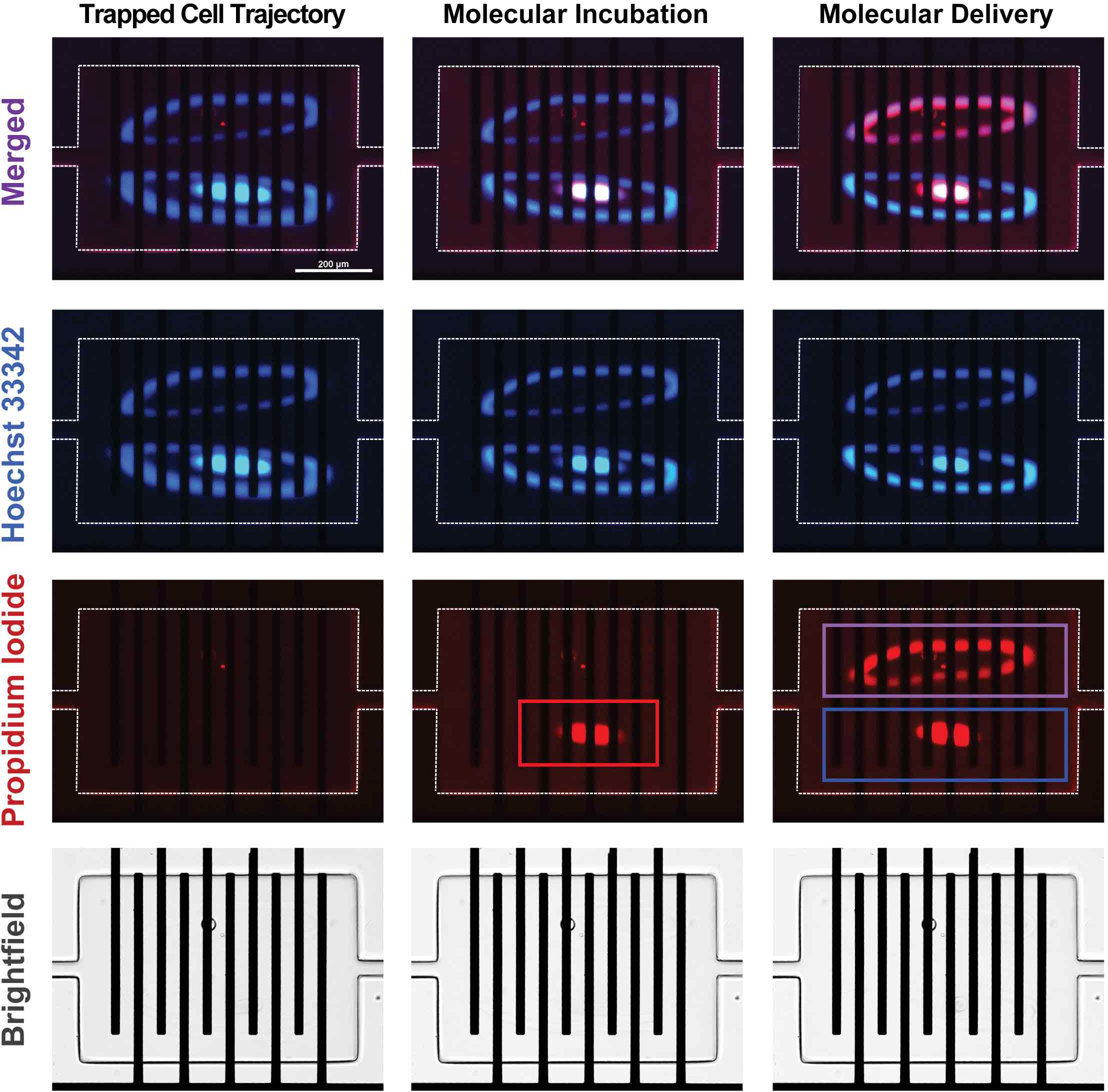

Supplement: S1 Fig — The white dashed lines outline the cell trapping chambers. Cancer cells (HCC827 GR6), pre-stained with live-cell nucleus marker Hoechst 33342, are isolated into cell-trapping microvortices. Cytosolic delivery via electroporation is visualized by detecting fluorescent signals of a membrane-impermeable dye Propidium Iodide (PI, 15 μM) from trapped cells. Fluorescent images of cells incubated in PI solution are taken before (Molecular Incubation) and after (Molecular Delivery) electroporation. Dead cells uptake PI prior to electroporation (red box). Successful electroporation is indicated by the overlap of PI and nucleus marker fluorescent signals (purple box). Cells that are not electroporated are indicated by the absence of PI fluorescent signals after electroporation (blue box). Scale bar = 200 μm. (TIF) [file pone.0264907.s003.tif]

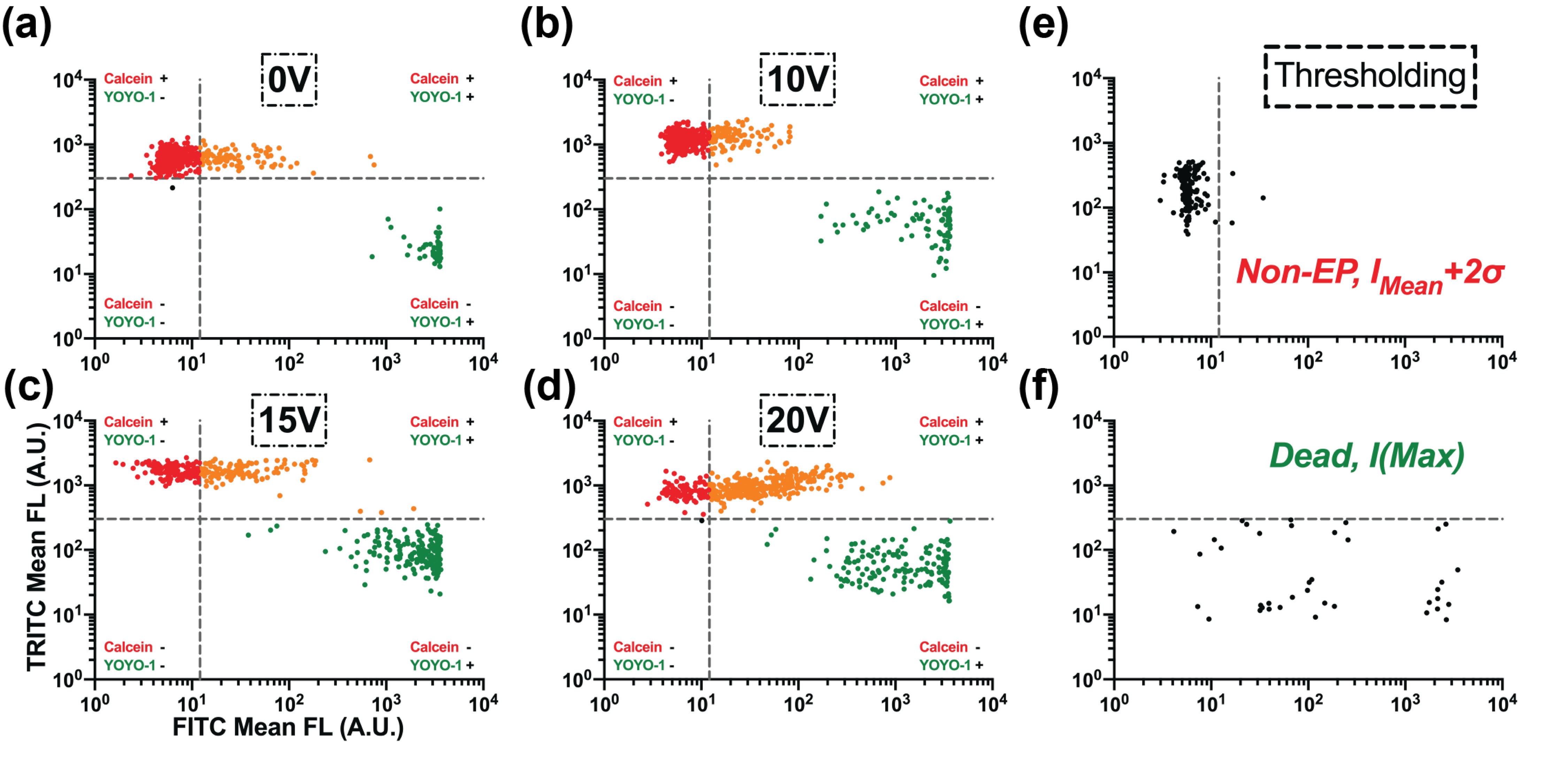

Supplement: S2 Fig — Generated 2D fluorescent intensity maps from each collected cell after electroporation in (a) 0V, (b) 10V, (c) 15V, and (d) 20V. Thresholding for determining successful electroporation (e) and cell viability (f) are described in the materials and methods section. Thresholding divides the intensity maps into 4 distinct quadrants, where the Calcein +/YOYO-1—quadrant represents viable but un-electroporated cells, Calcein -/YOYO-1 + quadrant represents dead cells presumably burst by the electroporation procedure, and Calcein +/YOYO-1 + quadrant represents viable and successfully electroporated cells. Calcein -/YOYO-1—quadrant represents cells that have fluorescent intensities too low to be considered for electroporation efficiency assessment. n > 150 cells for each intensity map from experiments in triplicates. (TIF) [file pone.0264907.s004.tif]

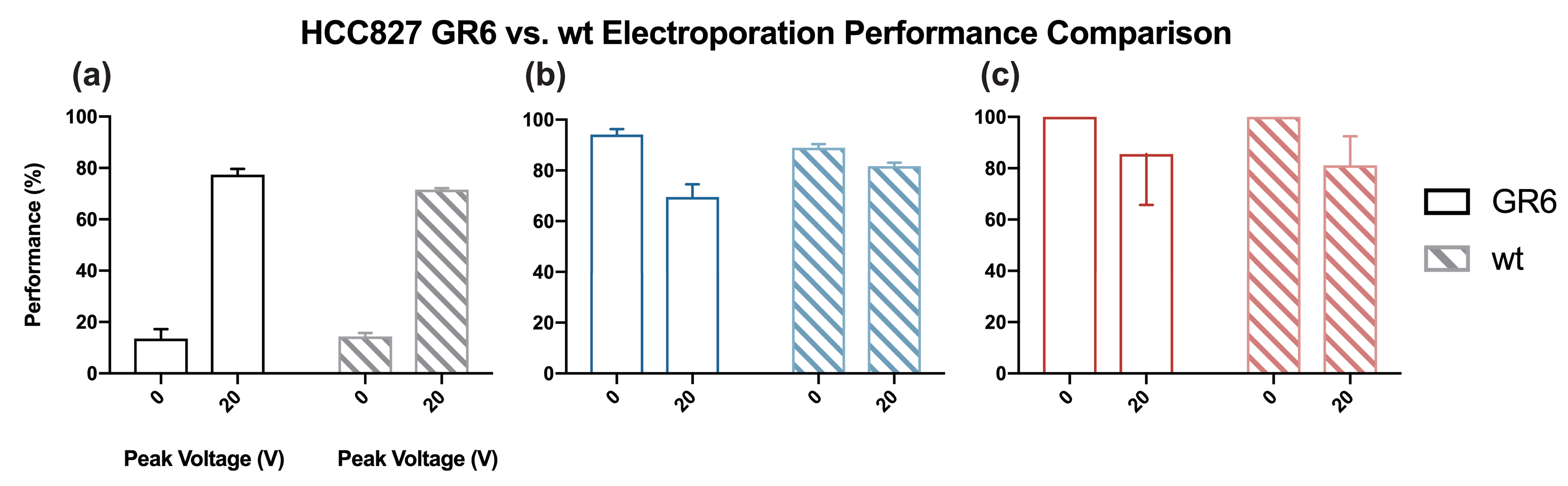

Supplement: S3 Fig — Efficiency (a, black), viability (b, blue), and collected cell count (c, red) are reported for GR6 (dark) and wt (light). The optimal voltage for successful electroporation for both HCC827 wt and GR6 variant was determined to be 20V due to consistent performance. Error bars represent standard error of mean (n = 3). (TIF) [file pone.0264907.s005.tif]

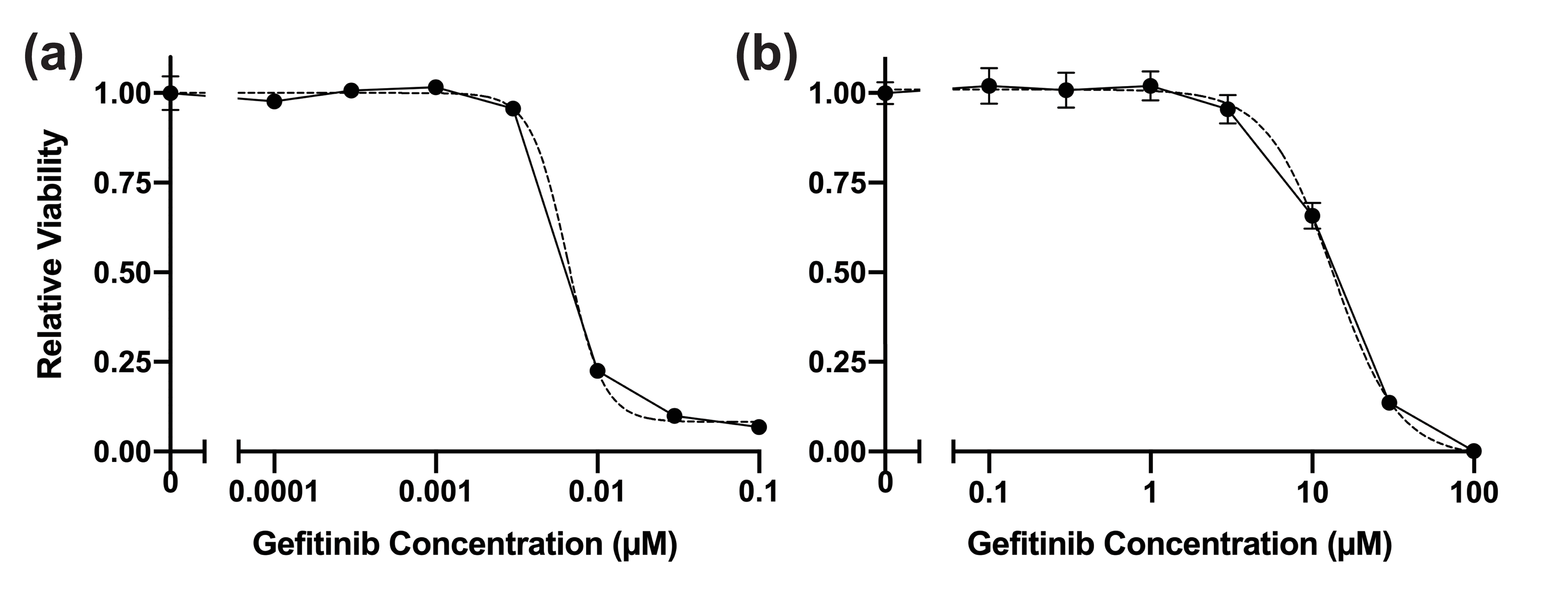

Supplement: S4 Fig — Cells (4000 cells/well) in conventional opaque assay well plates were treated with gefitinib at the indicated concentrations and measured for viability after 72 hours using the RealTime-Glo™ MT Cell Viability Assay Kit. Viability % is shown relative to untreated (DMSO) control wells. IC50 values were determined by using nonlinear regression analysis (variable slope, 4 parameter) and determined as the concentration where percent inhibition of cell viability relative to vehicle (DMSO) well is equal to 50%. Error bars represent standard error of mean (n = 3). (TIF) [file pone.0264907.s006.tif]

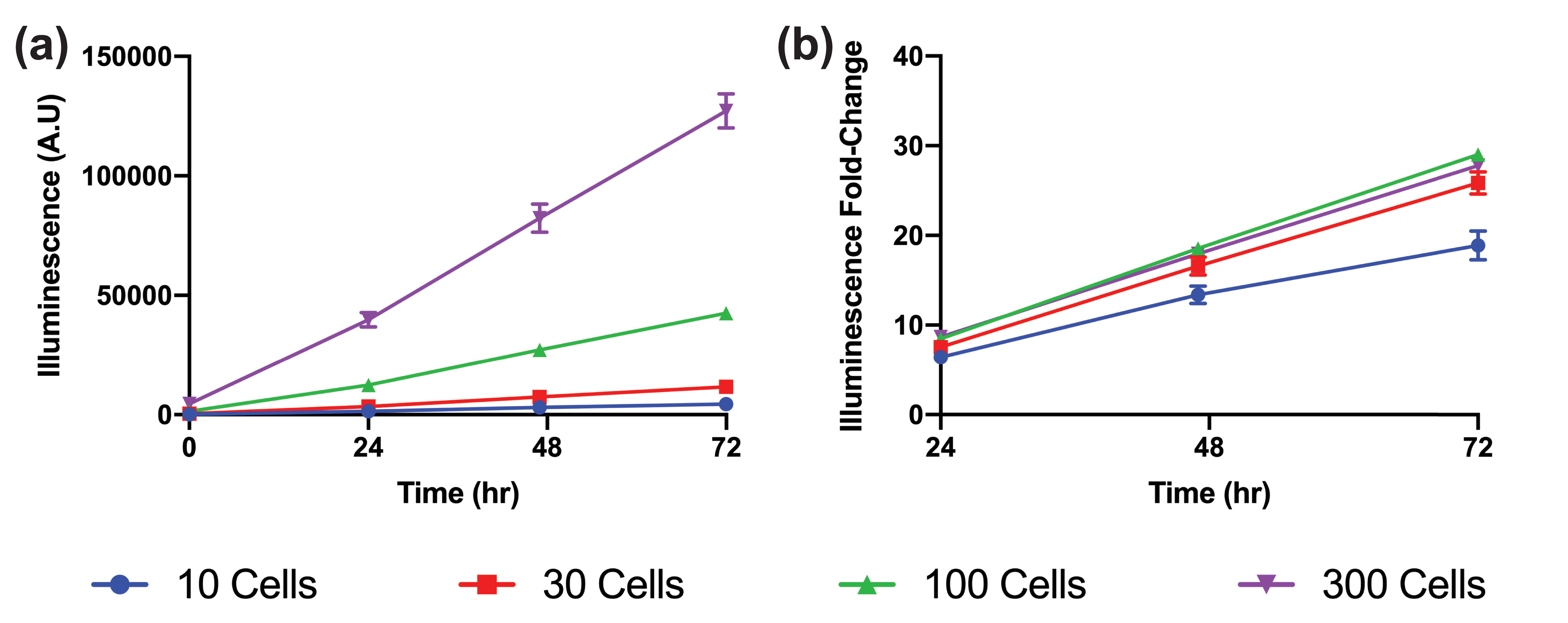

Supplement: S5 Fig — (a) HCC827 GR6 cells per well were plated on conventional 96-well assay plates and monitored for change in luminescence over time (0, 24, 48, 72 hrs). (b) Fold-change in luminescence linearly correlates over time, suggesting that the overall fold-change impact from various starting cell counts is negligible. A lower fold-change in luminescence, especially in low cell counts, could be attributed to difficulties in measuring low signals. Error bars represent standard error of mean (n = 3). (TIF) [file pone.0264907.s007.tif]
