# Supplementary material for: Sensitizing drug-resistant cancer cells from blood using microfluidic electroporator
Source: PLoS One. 2022 Mar 8;17(3):e0264907. doi: 10.1371/journal.pone.0264907 (PMC8903260; doi:10.1371/journal.pone.0264907)
Supplement: Attachment — Submitted filename: 2021_PONE-D-21-30672.docx [file pone.0264907.s008.docx]

Comments on # PONE-D-21-30672

Title: Sensitizing Drug-Resistant Cancer Cells from Blood using Microfluidic Electroporator

This manuscript presents a microfluidic system integrated with electrodes for generating vortex-assisted electroporation with the goal of developing routines allowing direct and active interrogation of CTCs directly from liquid biopsy samples. The system was validated by assessing drug response assays on wild-type (HCC827 wt) and gefitinib-resistant (HCC827 GR6) non-small cell lung cancer (NSCLC) cells. HCC827 cells trapped within microscale vortices were electroporated to sequentially deliver drug agents into the cytosol. Electroporation conditions facilitating multi-agent delivery were characterized for both cell lines using an automatic single-cell image fluorescence intensity algorithm. The proposed workflow, drug agent combinations to restore gefitinib sensitivity reflected the anticipated cytotoxic response. The system has the potential to enable functional interrogation of patient CTCs in situ, thereby accelerating the clinical standardization of liquid biopsies.

The study is of interest to the microfluidics and CTC community. The experiments are well designed and presented. I would like to recommend it to be published with the following comments addressed:

1. I have the impression that this study aims to develop a workflow that could allow direct assessment of patient samples. However, the study works with blood samples that are spiked with HCC827 wt and HCC827 GR6 cells. For proof-of-concept, spiking cells with the blood sample is reasonable. It may be better to make it clear that the workflow has not been able to work with patient sample directly yet and point out this would be the next step work.
2. It is not clear whether or not flushing the device with wash buffer prior to cell collection affects the processed cells, for example, are the cells flushed away?
3. When collecting the cells to 96-well plates, was the cell density controlled in each well? If yes, how? If not, would the cell density variation affects the study?
4. It is observed that 25V cause bubbles in addition to other problems. I believe 20V would cause bubble as well although it might not affect the study significantly. Please comment on that.
5. It seems the liquid sample with spiked cells is directly in contact with the electrodes. If this is correct, would the cells adhere to the electrodes quickly leading to unstable performance? Please comment whether or not a passivation layer designed on top of the electrodes to separate the liquid sample from the electrodes is possible to make the system work?
6. I suggest “a vacuum environment of 6e-6 torr” change to “a vacuum environment of 6^e-6^ torr”
